# Relationship between nine haloacetic acids with total organic halogens in different experimental conditions

**DOI:** 10.1186/1735-2746-10-26

**Published:** 2013-04-03

**Authors:** Hossein Pourmoghadas, Riley N Kinman

**Affiliations:** 1 Department of Environmental Science, Faculty of Agriculture and Natural Resources, Khorasgan (Isfahan) Branch, Islamic Azad University, Isfahan, Iran; 2 Department of Civil and Environmental Engineering, School of Engineering, University of Cincinnati, Cincinnati, Ohio 45221, USA

**Keywords:** Haloacetic acids, Total organic halogens, Total organic halogens, Haloacetic acids, Chlorination by-products

## Abstract

The effects of pH and bromide ion concentration on the formation of nine haloacetic acids (HAAs) and total organic halogens (TOX) in chlorinated drinking water have been evaluated. In an extensive study, the relationships of nine HAAs with TOX have been investigated. Honesty Significant Differences test (HSD) and ANOVA tests were used for the statistical analyses. The study determined the concentration range of nine HAAs as of a percentage of TOX at varying experimental conditions. Statistical analyses showed that the parameters pH and Br had significant effects on the formation of nine HAAs and TOX. This study also showed that brominated and mixed species of HAAs would be dominant in the presence of high bromide ion concentration which contributes a high percentage of the TOX. The results of this study could be used to set up a maximum contaminant level of TOX as a water quality standard for chlorination by-products.

## Background

Chlorination by-products (CBPs) are generally suspected of having adverse health effects. Toxicology studies have shown several CBPs including Haloacetic Acids (HAAs) to be carcinogenic or causing adverse reproductive or developmental effects in laboratory animals. Numerous epidemiological studies have suggested an increased cancer risk to individuals exposed to chlorinated waters [1,2]. According to animal studies DCA is believed to be a more potent carcinogen than THMs [3].

Similar to Trihalomethanes (THMs), HAAs are formed in the disinfection of drinking water with chlorine. It has been shown that, regardless of the source of the water, THMs and HAAs are the two largest classes of DBPs in treated drinking water [4,5]. The Maximum contaminant level for the total THMs is 0.080 mg/L and 0.060 mg/L for the sum of concentrations of five haloacetic acids (MCA, DCA, TCA, MBA, DBA ) To better control DBP in finished drinking water, it is important to understand the formation of DBP and speciation [4]. Since TOX could be used as a water quality standard, it is important to know the percentage of individual HAAs as the Total Organic Halogens (TOX) at varying experimental conditions.

Correlations between mutagenicity and water quality parameters are presented. The highest correlation was observed between mutagenicity and the total organic halide concentrations in treated samples [6]. Since bromide serves as a precursor in some of the organic by-products, which are brominated, and probably affects the formation of some of the non-halogenated by-products, it is important to understand its effect upon the formation of these by-products.

Bromide plays an important role in determining the relative concentrations of DBP species formed. It has been reported that in chlorinated water containing humic material, bromide has been shown to shift the distribution of THMs toward the more brominated and mixed halogenated species [4,5]. Bromide ion in surface and ground water could be increased by dibromoethane from diesel oil and gasoline, Br as an Impurity in the rock salt for deicing roads, geochemical, marine back ground contribution, mining, fertilizers, and pesticides [7].

The objective of this work was to determine the extent to which correlation exists between TOX and individual HAAs formation at varying experimental conditions. In this extensive study, the effects of the 3 pH values, 3 reaction times and 4 bromide concentrations on the formation of 9 HAAs: Monochloroacetic Acid (MCA), Dicholoroacetic Acid (DCA) Trichloroacetic Acids (TCA), Monobromoacetic Acid (MBA) Dibromoacetic Acid (DBA) and Tribromoacetic Acids (TBA) Bromochloroacetic Acid (BCA) and Dibromochloroacetic Acid (DBCA) and Dichlorobromoacetic Acid (DBCA) as percentage of TOX have been evaluated.

## Methods

Ultra pure water containing commercial humic acid (HA) with a nonvolatile total organic carbon (NVTOC) of 2.90 mg/L and a high chlorine dose of 25 mg/L was used as the principal model system. The independent variables were pH and bromide. The three levels of pH were 5, 7, and 9.4. The four bromide levels studied were 0.0, 0.5, 1.5, and 4.5 mg Br/L. The reaction times studied were 48 hours. All of the tests were conducted at 25°C. Table 1 shows the proposed Haloacetic Acid (HAAs) Target Compound List.

**Table 1:** Proposed Haloacetic Acid (HAAs) target compound list

	
Monochloroacetic Acid (MCA)	CH_2_CI-C0_2_H
Dichloroacetic Acid (DCA)	CHCI_2_-CO_2_H
Trichloroacetic Acid (TCA)	CCI_3_-CO_2_H
Monobromoacetic Acid (MBA)	CH_2_Br - CO_2_H
Dibromoacetic Acid (DBA)	CHBr_2_-CO_2_H
Tribromoacetic Acid (TBA)	CBr_3_ -CO_2_H
Bromochloroacetic Acid (BCA)	CHBrCl-CO_2_H
Dibromochloroacetic Acid (DBCA)	CBr_2_Cl-CO_2_H
Dichlorobromoacetic Acids (DCBA)	CCl_2_Br-CO_2_H

Factorial design was used to evaluate the experimental results statistically. A computer program (SAS) was used for the statistical analyses. The excel graphics software package was used for plotting the formation curves, and Lotus 1, 2, 3 was used for the calculations. Experimental procedures have been presented in detailed [4].

### Summary of the analytical procedures

a- Haloacetic acid determination: Liquid extraction with diethyl ether and esterification with diazomethane prior to GC-electron capture detector was used for the analysis of the nine HAAs. The EPA developed method detailed elsewhere [8].

b- Conversion Factors for the HAAs: Since the concentration of TOX is measured as chloride ion, in order to establish a relationship between individual HAAs and the TOX, the concentration of each HAAs was converted to chloride ion. The mass concentrations of DCBM, DBCM and TBM were converted to TCA and then converted to chloride ion. The following conversions were applied: BCA to DCA, MBA to MCAA and DBA to DCA. Table 2 shows factors used for conversion of HAAs to chloride ions. All chlorinated acetic acids (MCA, DCA and TCA) then were converted to chloride ions.

**Table 2:** Factors used for conversion of HAAs to chloride ions

**Mass oncentration of HAAs in μg/l**	**Conversion factor**
MCA as Cl	35.5 / 94.5
DCA as Cl	71 / 129
TCA as Cl	106.5 / 163.5
MBA as MCA	94.5 / 139
DBA as DCA	129 / 218
TBA as TCA	163.5 / 297
DCBA as TCA	163.5 / 208
DBCA as TCA	163.5 / 252.5
BCA as DCA	129 / 173.5

c- TOX determination: EPA method 450.1 [9] was utilized for the determination of TOX. This method consists of passing a sample of water (usually 40-100 mL) through a pair of minicolumns packed with 40 mg of granular activated carbon (GAC) previously milled and screened to 100-200 mesh. The minicolumns are mounted in series and the sample passes through the columns with an inert gas under pressure. Organic halides are removed from water by adsorption onto the activated carbon. After adsorption the carbon is washed with nitrate solution to remove interfering inorganic halide ions. The carbon is then transferred to a pyrolysis system in which the organic halides are combusted in a two step process that first converts the volatile components to the hydrogen halide for subsequent on-line titration with silver ion and measurement by microcoulmetry. All halide species are measured as chloride ions and are interpreted to represent a measure of carbon – absorbable organic halides (CAOX) which are usually considered to be a reasonable estimate of TOX in drinking water samples.

d- Statistical analysis: Factorial design method was used to statistically evaluate influences of different factors. Honesty Significant Difference test (HSD) and ANOVA tests were used for the statistical analyses.

## Results and Discussion

**Figure 1:**
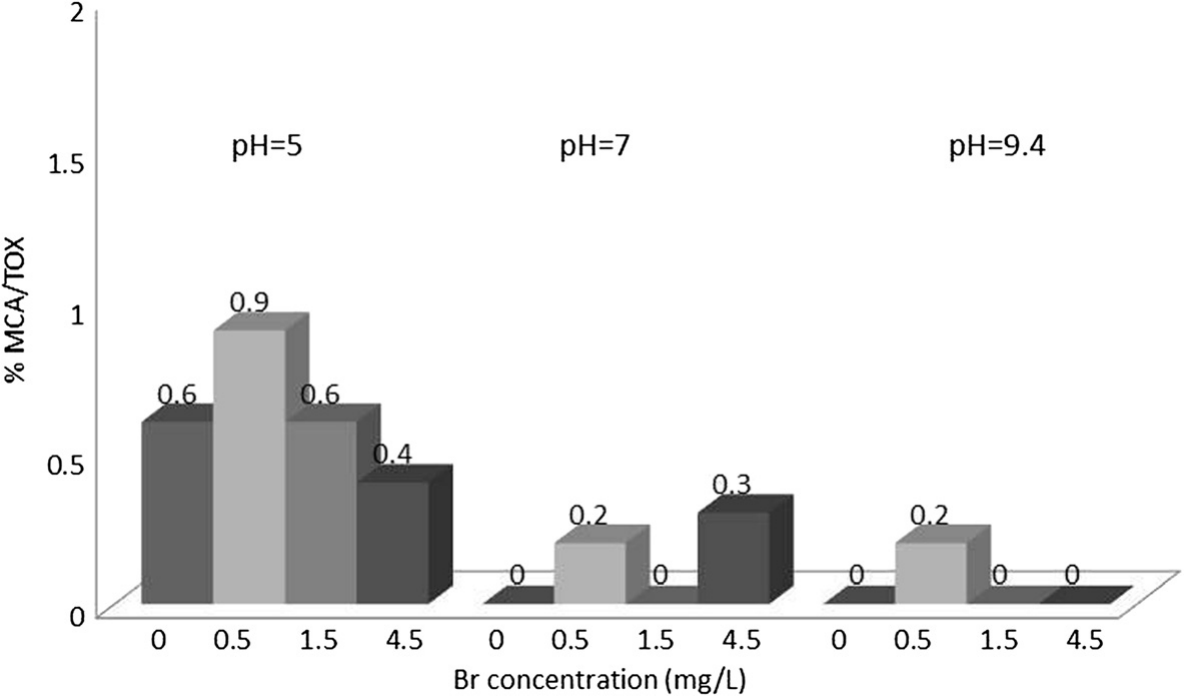
% TOX contributed by MCA; reaction time = 48 h.

**Figure 2:**
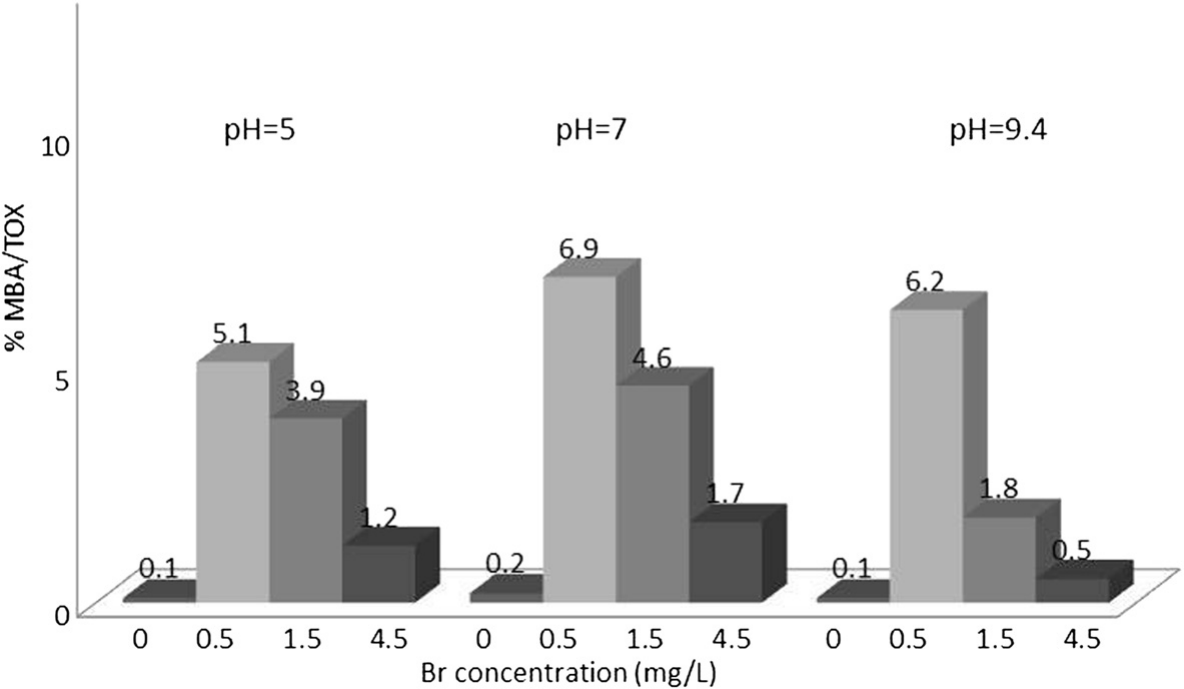
% TOX contributed by MBA; reaction time = 48 h.

**Figure 3:**
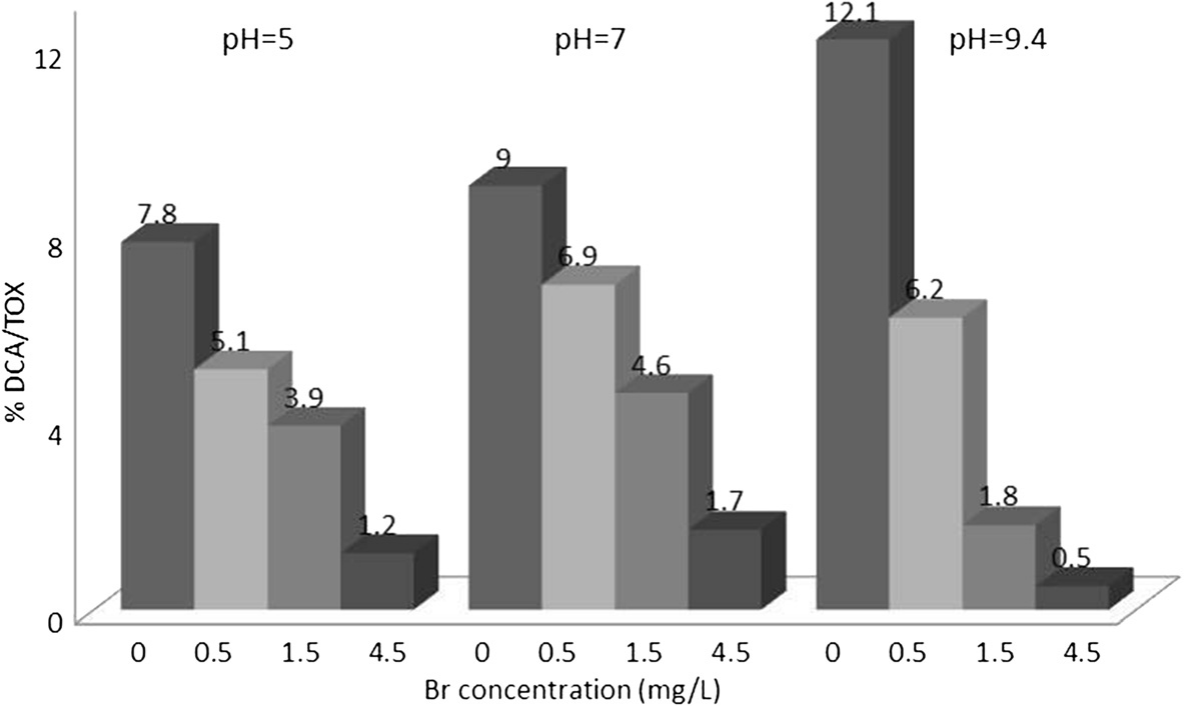
% TOX contributed by DCA;reaction time = 48 h.

**Figure 4:**
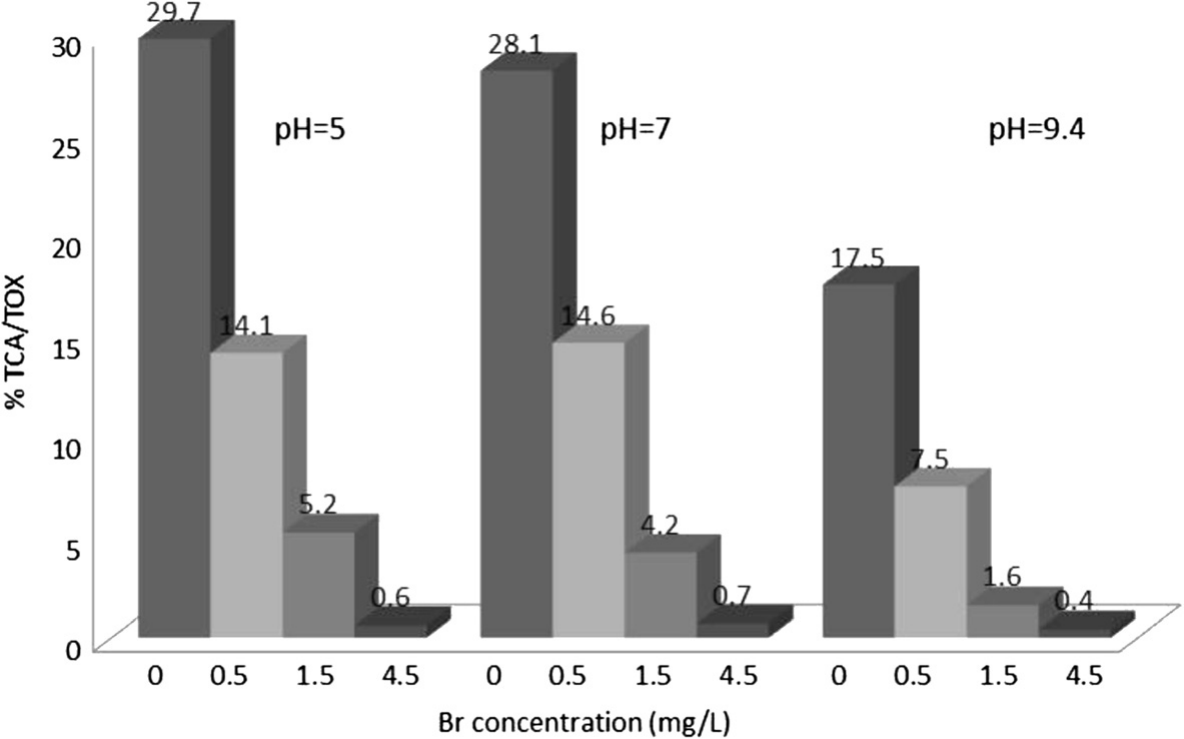
% TOX contributed by BCA; reaction time = 48 h.

**Figure 5:**
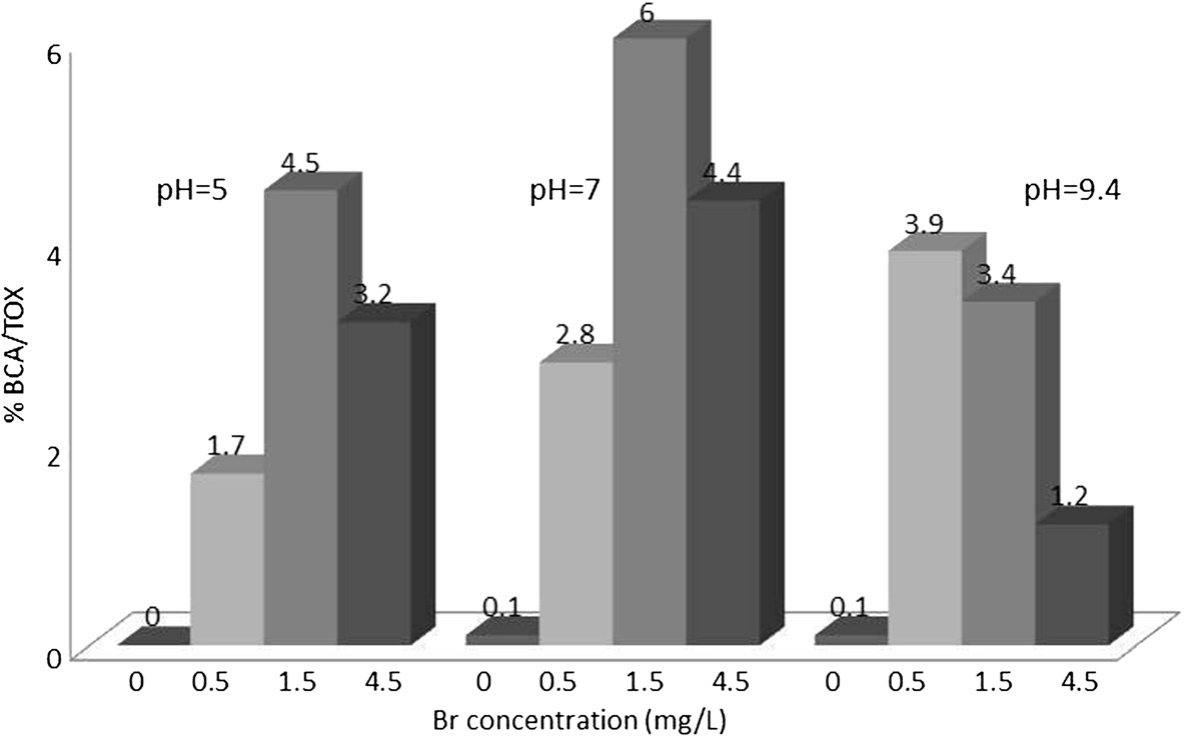
% TOX contributed by BCA; reaction time = 48 h.

**Figure 6:**
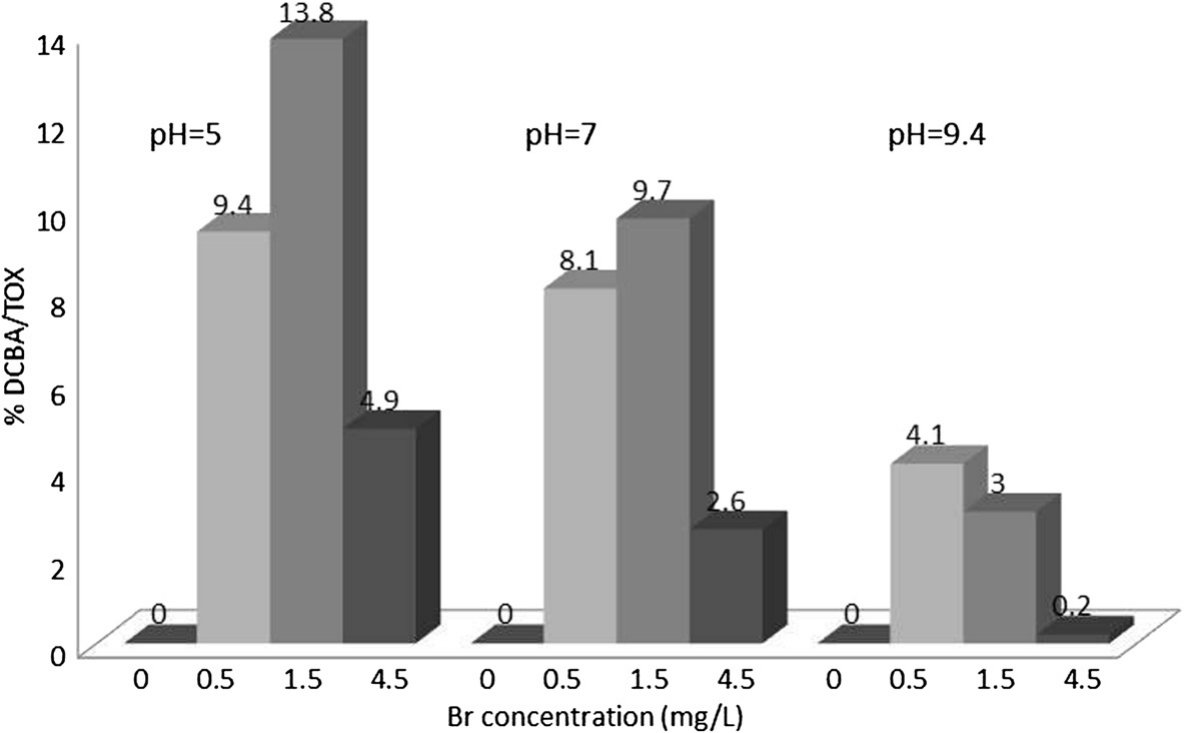
% TOX contributed by DCBA; reaction time = 48 h.

**Figure 7:**
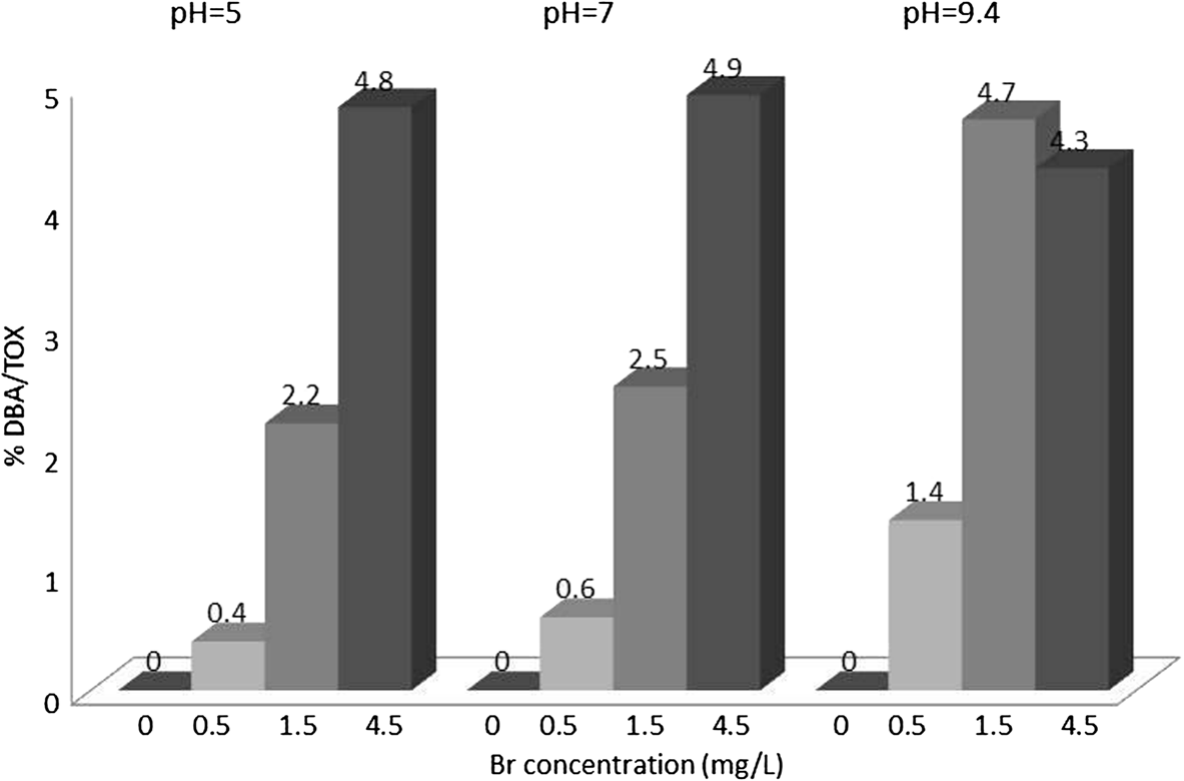
% TOX contributed by DBA; reaction time = 48 h.

**Figure 8:**
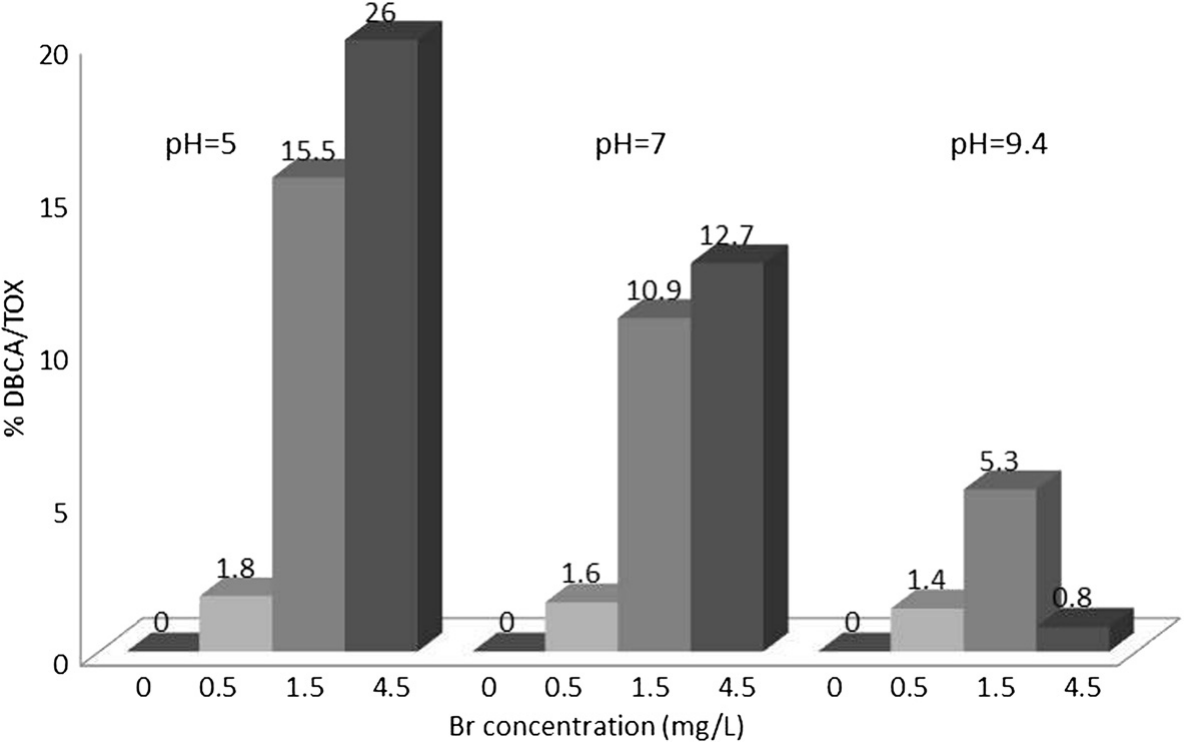
% TOX contributed by DBCA; reaction time =48 h.

**Figure 9:**
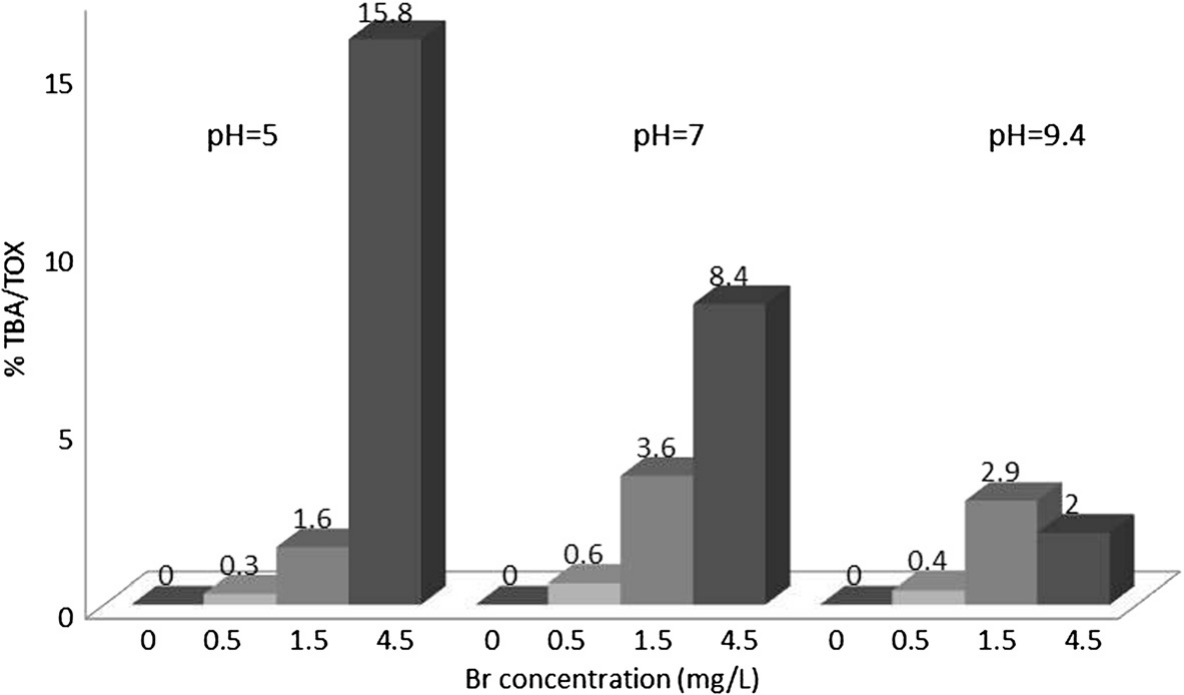
% TOX contributed by TBA; reaction time = 48 h.

The concentration of TOX was determined in all samples as chloride ions. The concentration of nine HAAs in μg/L were determined using liquid extraction, followed by GC determination. The concentrations of the HAAs in μg/L were converted to chloride ions. The contribution of individual HAAs to TOX (percent of TOX) was calculated, and plotted in Figures 1, 2, 3, 4, 5, 6, 7, 8, 9. Tables 3 and 4 show the results of statistical analyses, Honesty Significant Differences Test (HSD) and ANOVA tests for the TOX and individual HAAs.

**Table 3:** Results of honesty significant differences test (HSD)

	**TOX**	**MCA**	**MBA**	**DCA**	**TCA**	**BCA**	**DCBA**	**DBA**	**DBCA**	**TBA**
**Br Mg/L**										
0.0-0.5	N	S	S	S	S	S	S	S	S	N
0.0-1.5	N	S	S	S	S	S	S	S	S	S
0.0-4.5	S	S	S	S	S	S	S	S	S	S
0.0-4.5	S	S	S	S	S	S	S	S	S	S
0.5-4.5	S	S	S	S	S	S	S	S	S	S
1.5-4.5	S	S	S	S	S	S	S	S	S	S
**pH**										
5-7	S	N	N	N	S	S	S	S	S	S
5-9.4	S	S	N	S	S	S	S	S	S	S
7-9.4	S	S	N	S	S	S	S	S	S	N

S: Significant N: Not Significant P = < 0.05.

**Table 4:** Results of ANOVA test

	**TOX**	**MCA**	**MBA**	**DCA**	**TCA**	**BCA**	**DCBA**	**DBA**	**DBCA**	**TBA**
Br	S	N	S	S	S	S	S	S	S	S
pH	S	S	N	S	S	S	S	S	S	S
Hr	S	S	S	S	S	S	S	S	S	N
Br-pH	N	N	S	S	S	S	S	S	S	S
pH-Hr	N	S	S	S	S	S	S	S	N	S
pH-Br-Hr	N	S	S	S	S	S	S	S	S	N

S: Significant N: Not significant P = < 0.05.

### Monochloroacetic acid/TOX

Figure 1 shows the variation of percentage of MCA/TOX at different experimental conditions. On the average the percentage of TOX fluctuate at the 3 levels of Br and 4 levels of bromide concentration. It forms up to 0.9 percentage of TOX at pH = 5 and formation decrease to zero percent at pH values of 7 and 9.4.

### Monobromoacetic acid/TOX

Figure 2 shows the percentage of TOX contributed by MBA which is up to 6.9 percent at pH = 7 and 0.5 mg/L bromide concentration. At zero bromide level the percentage of MBA was expected to be zero, but this was not entirely the case, possibly because of the impurities of the reagents used, or analytical errors.

### Dichloroacetic acid/TOX

Figure 3 shows dichloroacetic acid as a percentage of TOX at different experimental conditions. In all 3 graphs the percentage of DCA contribution to TOX decreases by incremental addition of bromide. Highest percentage of TOX (12%) by DCA was observed at zero bromide concentration and pH= 9.4, and the reaction time of 48 hours. . The results of Tukey’s test (Table 3) show that in general, pH was a significant factor in the formation of DCA. Further statistical analysis ANOVA test (Table 4) revealed no significant differences between pH= 5 and pH= 7.

### Trichloroacetic acid/TOX

Figure 4 shows the percentage of TOX contributed by TCA. Similar to DCA the highest percentage of TOX was observed at zero bromide concentration for all different experimental conditions. The percentage of TOX contributed by TCA decreased by incremental addition of Br concentration, and higher pH values. In tables 3 and 4, it is shown that pH and Br had significant effect on the formation of TCA. At zero bromide concentration, 48 hours reaction time and pH =5 this haloacetic acid had the highest percentage (29.7%) of TOX.

### Bromochloroacetic acid/TOX

Figure 5 shows that the highest contribution to TOX was at the bromide concentration of 1.5 and pH = 7 (6%). Statistical analysis (HSD and ANOVA, Tables 3 and 4) show that the variable parameters had significant effects on the formation of BCA which affected the percentage of TOX. At pH =7, 9.4 and zero bromide level the percentage of BCA was expected to be zero, but this was not entirely the case, possibly because of the impurities of the reagents used, or analytical errors.

### Dichlorobromoacetic acid/TOX

Figure 6 shows the variation of DCBA/TOX at different experimental conditions. Bromide concentrations of 0.5 and 1.5 mg/ L produced highest DCBA concentration as a percentage of TOX. The highest percent of TOX was related to this haloacetic acid was 13.8% at lowest pH = 5 and 1.5 mg/L bromide concentration.

### Dibromoacetic acid/TOX

Figure 7 shows the percentage of this total organic halogen contributed by DBA with different experimental conditions. This ratio increased with the addition of bromide concentration, which was the highest at 4.5 mg/L Br. The highest percent was observed at pH = 7 and 4.5 mg/L. bromide concentration.

### Dibromochloroacetic acid/TOX

Figure 8 shows the lowest percentage of DBCA/TOX was observed at the pH = 9.4 for all bromide concentrations. The highest ratio was observed at bromide level of 4.5 at pH = 5 (26%).

### Tribromoacetic acid/TOX

Figure 9 shows the TBA as percentage of TOX from the chlorination of HA under different experimental conditions. The highest contribution observed was at Br concentration of 4.5 at all experimental conditions. The contribution of TBA to TOX increased up to 15.8 percent of TOX at 4.5 mg/L of Br ion, pH =5 and 48 hours reaction time. This was similar to TCA/TOX where the same trend was observed for TBA/TOX. Tables 3 and 4 show the results of statistical analyses, that almost all the parameters had significant effects on the percentage of TOX contributed by this haloacid.

## Conclusion

Statistical analyses (HSD and ANOVA Tests) showed that almost all the parameters Br, and pH had significant effects on the formation of individual HAAs and TOX. The study revealed that although TCA and DCA were the principal percentage of the TOX as HAAs, in the absence of bromide ion, these compounds decreased rapidly with the incremental addition of bromide.

This study also showed that brominated and mixed species of HAAs would be dominant in the presence of high Br concentration, which contributes to a high percentage of the TOX. The percentage of TOX contributed by individual HAAs depends on different experimental conditions. Bromide plays an important role in determination of relative concentrations of CBPs species formed. This investigation demonstrated that important parameters could change the percentage of HAAs compared to total halogenated by-products. Increasing bromide concentration of surface and ground water due to consumption of rock salt used for deicing the roads which contains bromide as impurity, diesel oil and gasoline which contain dibromoethane and marine back ground contribution could change the percentage of halogenated by-product from chlorinated to brominated ones. Overall the percentage of TOX by the nine HAAs decrease with increasing the pH values.

Accordingly, because the concentration of TOX is readily measurable, this parameter has become the focus of increased attention. TOX is a collective parameter that is being used increasingly as a surrogate for potentially harmful halogenated organic substances in drinking water [10]. Although TOX itself is currently not regulated in finished drinking water, good practice suggests that TOX formation should be controlled as well as THMs and HAAs formation. It has been reported that TOX formation tends to parallel THMs and HAAs formation. TOX could be determined easily in most countries all over the world as a reliable water quality parameter while it is not possible to determine the concentration of HAAs and THMs easily [11,12]. This information could help to determine TOX value for the maximum contaminant level for potable water quality to be used for a chlorination by-product water quality standard.

## Competing interests

Both authors declare that they have no competing interest.

## Authors’ contributions

HP Conception design, generation of data, assembly of data, analysis and interpretation of the data, drafting the manuscript. RNK supervisor of the research project, revision and approval of the manuscript. Both authors read and approved the final manuscript.
